# Reactivity to Measures of Metacognition

**DOI:** 10.3389/fpsyg.2019.02755

**Published:** 2019-12-06

**Authors:** Kit S. Double, Damian P. Birney

**Affiliations:** ^1^Department of Education, University of Oxford, Oxford, United Kingdom; ^2^School of Psychology, University of Sydney, Sydney, NSW, Australia

**Keywords:** metacognition, reactivity, judgments of learning, confidence ratings, think-aloud, cue utilization, judgments of learning, goal orientation

## Abstract

Metacognition is typically measured by collecting self-reported information from participants while they complete a cognitive task. Recent evidence suggests that eliciting such metacognitive information from participants can impact both their metacognitive processes and their cognitive performance. Although there are contradictory findings regarding the magnitude and even the direction of this effect, recent evidence has converged to provide a clearer picture of the mechanisms that determine reactivity. Here, we provide a review of the evidence that measures of metacognition, namely think-aloud protocols, judgments of learning, and confidence ratings, are reactive. We argue that reactivity has important implications not just for the measurement of metacognition, but for metacognition theorizing because reactivity can provide insights into the cues participants use to monitor their performance. Drawing from this synthesis of evidence, we propose a tentative framework for studying reactivity that integrates cue processing accounts of reactivity with existing models of metacognition. We conclude the review by addressing some of the pertinent questions yet to be comprehensively addressed by reactivity research, including how researchers should best address issues of reactivity when using experimental designs.

## Introduction

Broadly speaking metacognition refers to the knowledge and regulation of one’s own cognition (Veenman et al., 2006). At its highest level, the conceptualization of metacognition is frequently broken into two discernible processes – a monitoring process and a control process (Flavell, 1979) – although many more specific processes within the concept of metacognition can be distinguished (Tarricone, 2011). The ability to monitor and control one’s own cognition effectively is vital to cognitive performance and everyday function. Metacognition is crucial in the performance of many cognitive processes, including: error monitoring (Yeung and Summerfield, 2012), regulating learning (Efklides, 2011), allocating cognitive resources (Son and Metcalfe, 2000), and strategy selection (Karpicke, 2009).

Metacognitive monitoring allows an individual to detect errors and allocate resources effectively (Carter et al., 1998). Theories of metacognition regard monitoring as central to an individual’s ability to regulate their own thinking and behavior (Nelson and Dunlosky, 1991). In particular, metacognitive monitoring is vitally important when learners make study time decisions (e.g., Son and Metcalfe, 2000; Metcalfe and Finn, 2008). Furthermore, learners need to regularly evaluate the effectiveness of different study activities on their learning in order to select the best possible study behaviors (Flavell, 1979). Metacognitive monitoring is typically measured using item-level self-report measures, including judgments of learning (e.g., Baldi, 1996; Castel et al., 2007; Baumeister et al., 2015; Dunlosky et al., 2015) and confidence ratings (e.g., Stankov and Crawford, 1996; Stankov, 2000; Stankov and Lee, 2014a). These measures have provided significant insights into the calibration of individuals’ metacognitive monitoring (Stankov, 1998, 2000) and how such judgments influence subsequent behaviors (Metcalfe and Finn, 2008). However, until recently, little attention had been paid to the effect that eliciting such measures has on participants’ underlying cognitive performance.

### Reactivity

A significant concern for psychology is the potential for participants to react in some way to the fact that they are being measured. A number of these effects have been observed and researched, including the Hawthorne Effect (when participants alter their behavior due to researcher attention; see McCarney et al., 2007), the John Henry Effect (where the control group alters their behavior due to an awareness of being in the control group; see Saretsky, 1972), and more generally the observer-expectancy effect (where experimenters subtly communicate their expectations to participants and they alter their behavior to meet these expectations; see Rosenthal, 1966). These effects are examples of a phenomenon known as *reactivity*. Reactivity refers to instances where individuals modify their behavior or performance in response to being measured or observed. Participants can react to being measured in a variety of ways, including changes in motivation, expectations, affect, or goals. Reactivity to measurement has been examined within a large number of domains, including health behaviors (French and Sutton, 2010), purchasing intentions (Morwitz and Fitzsimons, 2004), and medicine (Colagiuri et al., 2013). Reactivity to measurement has also been used to promote behavior change. For example, it has been used as an intervention to reduce smoking (McCarthy et al., 2015) and increase exercise (Izawa et al., 2005). Generally speaking, being measured appears to increase positive behaviors, while reducing negative behaviors (e.g., Kazdin, 1974).

When self-report measures are collected “online,” that is while a participant is performing a task (e.g., item-by-item confidence ratings), there is an even greater risk of reactivity. As such, self-report measures such as those used to assess metacognition, may be particularly susceptible to reactivity effects (Mitchum et al., 2016). In a cognitive task, performing a self-report measure can either facilitate performance, so-called *positive reactivity*, or alternatively it may impair performance, referred to as *negative reactivity*. Given the widespread use of “online” self-report measures to assess metacognitive processes, reactivity is a significant concern for metacognitive research because it implies the ratings themselves may never be truly accurate because they are influencing the process they are designed to measure.

Here, we present a review that examines the evidence that measures of metacognition typically used in the research literature are reactive. While recent meta-analyses have provided some clarity as to the overall size of reactivity effects (Fox et al., 2011; Rhodes and Tauber, 2011; Double et al., 2018), we aim to provide a more nuanced review focusing on the variables that determine the conditions under which reactivity does and does not arise. To this end, we will extend previous theories concerning the mechanisms of reactivity by incorporating recent advances in the field and integrating findings from both experimental and individual differences perspectives on reactivity. This is in order to provide a framework for the study of reactivity that integrates person-level and environmental variables. Finally, we will argue that understanding reactivity can provide insights into the manner and mechanisms in which participants monitor their performance including which cues they do and do not draw upon when performing self-report measures of reactivity.

We will first review the evidence for reactivity in the most common measures of metacognition: think-aloud protocols, judgments of learning, and confidence ratings. We will then review current theoretical frameworks of reactivity before proposing a new tentative framework that aims to bring together this research to help promote a greater understanding of reactivity. Next, we will discuss the implications and issues that are raised by reactivity research. Finally, we will assess what researchers can do to minimize reactivity effects.

## Evidence for Reactivity to Measures of Metacognition

### Think-Aloud Protocols

Think-aloud protocols involve participants verbalizing their internal cognitive processes in an online fashion (Fox et al., 2011). While think-aloud protocols are somewhat more involved than eliciting confidence ratings or judgments of learning (JOLs) to measure metacognition, think-aloud protocols are a typical way that researchers measure participants’ subjective experience of cognition and consciousness, and are often specifically viewed as an assessment of metacognition (e.g., Berardi-Coletta et al., 1995; Bannert and Mengelkamp, 2008). Reactivity to think-aloud protocols has been a topic of considerable research, at least compared with other methods for measuring metacognitive processes and this research has been important in the development of theories of reactivity. In a comprehensive meta-analysis of 94 studies, Fox et al. (2011) found that think-aloud procedures that do not demand additional information from a subject (i.e., they require only that the subjects vocalize their current cognitions) were not reactive, whereas protocols that directed subjects for additional information, such as to provide explanations for their thought processes, displayed positive reactivity.

Although the average reactivity effect reported in the above meta-analysis indicates that think-aloud protocols are unlikely to be reactive unless they demand additional information from individuals, there is some evidence that this may depend on the task. Fox and Charness (2010) found that performance on Raven’s Progressive Matrices (a measure of fluid intelligence) was particularly reactive to think-aloud procedures in older participants (but not younger participants), even when no additional information was sought from the participants. In fact, the think-aloud group gained an average of 11 IQ points. Performance on other tasks (paired-associates, cube comparison, and mental multiplication), however, was not affected by performing the think-aloud protocol. The authors tested a hypothesis that think-aloud procedures helped older adults inhibit the processing of irrelevant information but found no evidence for such a process. As such the mechanism by which think-aloud protocols benefit problem-solving performance, along with the extent to which these findings generalize to participants of other ages, other tasks, or other measures of metacognition, is as yet unclear.

### Judgments of Learning

Judgments of learning (JOLs) typically require participants to evaluate the likelihood that they will recall a recently learnt word-pair on a later test. JOLs are typically elicited in word-pair or word list learning paradigm and can either be elicited immediately after presentation of the to-be-learned stimuli or after a delay. Delayed JOLs tend to be more accurate than JOLs collected immediately after study (Nelson and Dunlosky, 1991). The evidence for reactivity to JOLs is so far equivocal. A majority of studies observed positive reactivity (Zechmeister and Shaughnessy, 1980; Dougherty et al., 2005; Soderstrom et al., 2015; Witherby and Tauber, 2017; Janes et al., 2018); however, others have found no reactivity (Kelemen and Weaver, 1997; Benjamin et al., 1998; Tauber and Rhodes, 2012). While a recent study by Mitchum et al. (2016) found negative reactivity.

Mitchum et al. (2016) directly investigated reactivity to JOLs in a series of five experiments. They demonstrated negative reactivity to JOLs for unrelated word-pairs when the series of presented word-pairs contained both related and unrelated pairs, but not if only unrelated pairs were presented. They suggested that eliciting JOLs prompted participants to prioritize studying the easier (related) word-pairs in order to maximize their performance, what they referred to as the *changed goal hypothesis*, which we will return to later. As evidence of this hypothesis, Mitchum et al. (2016) found reactivity only occurred when study time was participant-paced not experimenter-paced. They argued that this suggests that eliciting JOLs prompts participants to modify their time spent learning related vs. unrelated word-pairs.

In contrast, using a similar paradigm, Soderstrom et al. (2015) found that performing JOLs resulted in better recall of related pairs, but had no effect on unrelated pairs. They hypothesized that performing JOLs enhances recall for the information used in making the judgment, which in the case of related word-pairs was the relatedness of the cue. Given that the relationship between the cue and target is informative when participants come to the criterion test, performance on related word-pairs is facilitated. Janes et al. (2018) performed a further investigation of this effect, which they dubbed the *increased relatedness effect*, to try and examine whether JOLs improved performance on related word-pairs, impaired performance on unrelated word-pairs, or both. Their results suggested that the increased relatedness effect was largely driven by positive reactivity in related word-pairs.

Other studies have found that JOLs are not necessarily reactive (Kelemen and Weaver, 1997; Benjamin et al., 1998; Tauber and Rhodes, 2012). Perhaps most notably, in an attempt to replicate an early finding of reactivity, Dougherty et al. (2018) demonstrated four experiments where reactivity was not observed. However, all four experiments utilized unrelated word-pairs, which appear to be less prone to reactivity (see below for a discussion of potential reasons). In a meta-analysis of JOL reactivity, Double et al. (2018) examined the effect of eliciting immediate JOLs compared with no judgment controls. Supporting, the above experimental findings, they found that there is a small but significant positive effect of performing JOLs on recall performance when word-pairs are related, but no effect when word-pairs are unrelated. However, this finding was somewhat obscured by the large heterogeneity in the *direction* of observed reactivity effects as well as the small number of studies that had examined reactivity to immediate JOLs.

A meta-analysis by Rhodes and Tauber (2011) further examined reactivity to JOLs by comparing recall performance when delayed judgments of learning were elicited versus immediate JOLs. Analyzing 98 effect sizes, they found a small (*g* = 0.08) positive effect of eliciting delayed compared with immediate JOLs on recall performance. This finding suggests that delayed JOLs may be more prone to reactivity than immediate JOLs. Given that delayed JOLs tend to be more accurate than immediate JOLs and are presumably based on more diagnostic information than immediate JOLs, Rhodes and Tauber (2011) concluded that reactivity was driven by the increased processing of this diagnostic information (i.e., criterion-test relevant cues) when delayed JOLs are elicited.

### Confidence Ratings

Confidence ratings (CR) are one of the most frequently used measures of metacognition (Stankov, 1998, 2000; Stankov and Lee, 2008; Fleming and Lau, 2014; Yeung and Summerfield, 2014) and have been integrated into a range of study domains including intelligence (e.g., Stankov, 2000), learning paradigms (e.g., Don et al., 2016), reasoning tasks (e.g., Crawford and Stankov, 1996; Stankov, 2000), decision-making (Jackson and Kleitman, 2014), and perceptual tasks (Stankov, 1998). While JOLs are typically utilized in memory research, where the goal is to encode and recall novel stimuli, CR are typically utilized in either complex reasoning tasks (e.g., fluid intelligence measures) or simple perceptual discrimination tasks. The focus of reactivity has largely been with respect to complex reasoning tasks. Metacognitive monitoring in reasoning tasks has a similar global architecture to monitoring within memory paradigms, but with additional components that account for the complexity of the cognitive processes unique to reasoning (Ackerman and Thompson, 2017). This process is more temporally dependent, with participants moving from intermediate theories of confidence toward a subjective confidence criterion as evidence for a particular response accumulates (Brown and Heathcote, 2008; Ackerman, 2014). It is pertinent to compare the evidence for reactivity in JOLs and CR, while acknowledging this is somewhat confounded by the fact that these ratings are generally used with memory/reasoning tasks respectively, which often have distinct cognitive and metacognitive processes (Ackerman and Thompson, 2015).

Double and Birney (2017a) performed a direct experimental test of reactivity to CR in a student population. Our first experiment compared performance on Raven’s Progressive Matrices between participants who provided CR after each trial with a control condition who did not provide ratings. The results indicated that participants who provided CR performed better overall on Raven’s Progressive Matrices than controls, with no observed differences in response time. A second experiment (in a sample of older adults) attempted to distinguish between two hypothesized mechanisms for this effect – the cognizant confidence hypothesis and the general cognitive benefit hypothesis. The defining distinction between these two proposed mechanisms was the fact that the cognizant confidence hypothesis predicted that only confident participants would benefit from providing CR, because eliciting CR was activating pre-existing self-confidence beliefs. We had participants perform a battery of reasoning tasks, designed to broadly assess their reasoning abilities, as well as a self-report measure of their reasoning self-confidence. After these initial measures, participants performed Raven’s Progressive Matrices either with or without CR. The results indicated that performing CR facilitated the performance of high self-confidence participants, but impaired the performance of low self-confidence participants, and this result held even when baseline reasoning ability was controlled for. These results were tentatively interpreted as supporting the idea that eliciting CR activates pre-existing self-confidence beliefs.

In a more recent study of CR reactivity, Double and Birney (2019) examined the extent to which reactivity can be accounted for by priming compared with genuine metacognitive introspection. In a first experiment, they showed that reactivity occurs, and is moderated by self-confidence, both in traditional CR and in a condition where participants performed task-irrelevant CR (i.e., rating their confidence that two squares were the same color). However, reactivity did not occur when participants made task-irrelevant ratings but the word “confident” was absent. A second experiment expanded this finding by demonstrating that reactivity effects were somewhat negated when the word “confident” was replaced with the word “likely.” Together these findings suggested that the specific wording of CR can be important in determining the magnitude of reactivity effects. Specifically, it may be that the inclusion of the word “confident” in CR directs participants’ attention toward their confidence-related beliefs and thus drives the moderation of reactivity by self-confidence.

Like JOLs, however, there have also been a number of studies that have shown that CR are not always reactive (Ackerman and Goldsmith, 2008; Thompson et al., 2013; Ackerman, 2014). For example, Ackerman and Goldsmith (2008) found no effect of CR elicited during general knowledge questions. Similarly, Ackerman (2014) found little differences in performance on complex remote associate tasks between experiments where intermediate CR were elicited. This is surprising given that one might predict the effect of intermediate CR to be greater than retrospective CR. However, the tasks used in these studies differ substantially from those where reactivity to CR has been demonstrated, i.e., fluid reasoning tasks (Double and Birney, 2017a, 2018, 2019). This might suggest that fluid reasoning tasks are particularly prone to reactivity effects as suggested by Fox and Charness (2010). Additionally, given that both Ackerman and Goldsmith (2008) and Ackerman (2014) manipulated the presence of CR across experiments (rather than within), more direct replications with respect to reactivity may also be needed.

The extent to which eliciting CR affects metacognitive monitoring has also been explored. Double and Birney (2018) had participants provide predictions about their performance on a reasoning task both before and after they competed the task. The retrospective appraisal at the end of the task was used to assess participants’ overall monitoring of performance. Aggregate judgments typically align well with item-level judgments, although they show less over-confidence (Gigerenzer et al., 1991; Schneider et al., 2000). Participants who performed CR during the task made significantly *less* accurate retrospective appraisals of their performance compared to a control group who did not provide CR. There was also a stronger relationship between the pre-task prediction and the post-task prediction for the CR group, suggesting that eliciting CR led to increased attention on pre-existing beliefs and less focus on actual performance. Arguably this finding suggests that, at least for CR, eliciting metacognitive measures may actually disrupt the metacognitive monitoring process because providing CR directs attention to pre-existing beliefs and away from actual performance, leading to less accurate monitoring.

In one of the only studies to examine reactivity to CR in a more simple perceptual task, Petrusic and Baranski (2003) found that eliciting CR resulted in slower decision response times using a perceptual choice paradigm. Furthermore, while they found that CR did not affect accuracy, they noted that error rates were higher on 80% of the stimuli when CR were elicited and the lack of significance may have been be due to a lack of power. In a later set of studies, Birney et al. (2017) compared performance across two studies of industry managers, one where they included CR in Raven’s Progressive Matrices and one where they did not. They found that performance was lower in the study where CR had been elicited. Additionally, the impact of CR appeared to interact with difficulty, such that performance on easy items was higher when CR were elicited, but performance on difficult items was impaired.

## Theoretical Frameworks of Reactivity

### Prompts and Instructions

Reactivity is often explained as a by-product of changes in participants’ attention (Ericsson and Simon, 1993). Reactivity occurs when the self-monitoring or self-evaluation prompted by self-reporting metacognitive processes leads participants to attend to internal cognitions in a way they would not have ordinarily done. This is particularly the case for think-aloud protocols, which often involve the added effects of having one’s verbal responses recorded, as well as the presence of an experimenter. As such researchers performing think-aloud protocols have often been encouraged to minimize experimenter prompts (other than to remind the participant to continue talking), and to design prompts that do not encourage self-reflection or self-evaluation (Ericsson and Simon, 1993). This view of reactivity argues that self-report measures of metacognition can reliably be used if the researcher can reduce the self-reflective demands on the participant to that which would ordinarily be performed.

### Cue Utilization/Processing

Building on this approach, reactivity to metacognitive ratings has often been explained in terms of the cues that ratings (JOLs and CR) prompt participants to attend to. Koriat’s (1997) dual-basis view of metacognitive ratings delineates between experience-based cues and theory-based cues. Experience-based cues refer to cues used to make JOLs that are drawn from the actual experience of solving or memorizing an item, e.g., “How quickly did I solve that problem?” Information-based cues, on the other hand, refer to cues that are drawn from pre-existing beliefs about one’s competence, e.g., “Do I have a very good memory?” Metacognitive monitoring within memory tasks tends to rely on experience-based cues such as ease of processing or retrieval fluency (Begg et al., 1989; Benjamin et al., 1998; Tauber et al., 2019). However, when asked to provide a metacognitive rating, participants will draw on whatever cues are salient. For example, as mentioned, including the word “confident” in CR may make participants’ pre-existing beliefs about their self-confidence salient (Double and Birney, 2019). Often this account has been used to explain positive reactivity – with additional processing of cues when making a metacognitive rating improving performance if a criterion test utilizes the same cues. For example, Soderstrom et al. (2015) argued that positive reactivity is observed in related word-pairs because participants spend additional cognitive resources processing the *relationship* between the cue and the target when making their JOL. This account also explains why reactivity may be more likely when related and unrelated word-pairs are presented as part of the same list. When related and unrelated word-pairs are presented concurrently, compared to pure lists of related word-pairs, the relatedness of the related word-pairs is more salient due to the contrast with the unrelated word-pairs (Mitchum et al., 2016; Janes et al., 2018). There is further indirect evidence that eliciting metacognitive self-reports can make judgment-relevant cues more salient. Koriat and Ackerman (2010) found that when monitoring a peer’s performance, participants do not ordinarily rely on the same heuristics and cues as they do when monitoring their own (e.g., effort). However, if they first monitor their own performance and then a peer’s, they subsequently utilize the same cues for evaluating the peer as themselves. This suggests that eliciting the judgments during metacognitive monitoring of one’s own performance makes judgment-relevant cues more salient when monitoring a peer’s performance.

Metacognitive monitoring within reasoning tasks is similarly based on inferences from available cues (Ackerman and Thompson, 2017). While less is known about the cues utilized during metacognitive monitoring during reasoning, similar distinctions between monitoring of the momentary experience (e.g., perceptual fluency) and monitoring of global information about one’s abilities have been made (Ackerman, 2019). While the cues used to make metacognitive judgments in memory and reasoning paradigms are largely shared, there are also some distinctions. The fact that reasoning tasks tends to be more cognitively complex may mean that task characteristics play a more important role in metacognitive judgments during reasoning (Ackerman, 2019). Furthermore, monitoring of reasoning tasks tend to draw more heavily from information-based cues and self-perceptions (Stankov, 1998; Double and Birney, 2018; Ackerman, 2019). The self-perceptions used to inform CR are varied, and include such factors as need for cognition (Cacioppo and Petty, 1982), math anxiety (Legg and Locker, 2009), and self-efficacy (Double and Birney, 2017b). Many different self-perceptions will be utilized when making CR and will therefore have an important role in determining the direction and magnitude of reactivity.

### Goal Orientations

Measures of metacognition often require some form of self-assessment and many ratings are used to form indices of calibration or bias (Fleming and Lau, 2014). Self-evaluation often results in goal-directed behavior as individuals become aware of discrepancies between their current behavior and their goals (Silvia and Duval, 2001). Mitchum et al. (2016) put forward the hypothesis that performing JOLs causes individuals to shift to a performance-orientation and away from a mastery-orientation because of this requirement to self-assess. Additionally, Double and Birney (2018) found that participants who perform CR tended to focus on short-term performance, with CR improving mean performance on a timed reasoning task (when only the attempted items were considered), but not overall performance. They argued that CR prompted participants to become more performance orientated by focusing on getting the item in front of them correct rather than getting through as many items as possible within the time limit. Finally, Double and Birney (2017b) found that participants who provided CR performed better than controls on a reasoning test, but performed worse on a surprise test of the rules used in the task. They argued that CR prompted participants to focus on performing well on the task (memorizing the rules was unnecessary as they were present on screen), rather than mastering the content of the task.

## A Cue-Driven Metacognitive Framework of Reactivity

Below, we provide a tentative framework for the study of reactivity that attempts to incorporate the perspectives outlined above. Importantly, our goal is not to provide an overview of every variable that could influence the magnitude and direction of reactivity, but rather to incorporate these findings regarding reactivity with existing frameworks for the study of metacognition and self-assessment, particularly cue utilization theory. This framework is intended to encourage additional empirical research and derive testable predictions rather than provide a complete overview of reactivity effects.

This model starts with the basic tenant of cue utilization theory, that there is a distinction between information-based cues and experience-based cues (Koriat et al., 2008). Under these two broad umbrellas, this framework also integrates cues put forward by theories of cue utilization within meta-reasoning such as self-perceptions and task characteristics (Ackerman, 2019). Drawing from this framework, the model proposes that (1) there is a pool of potential cues, some of which are information based and some of which are experience based (Koriat et al., 2008); (2) if attended to, these cues become salient and inform the metacognitive monitoring process (e.g., Koriat and Ackerman, 2010); and (3) both salient and non-salient cues can influence cognitive performance, for example, even if an individual does not attend to a cue (e.g., difficulty), the cue still may be having an effect on cognitive performance.

**Figure 1:**
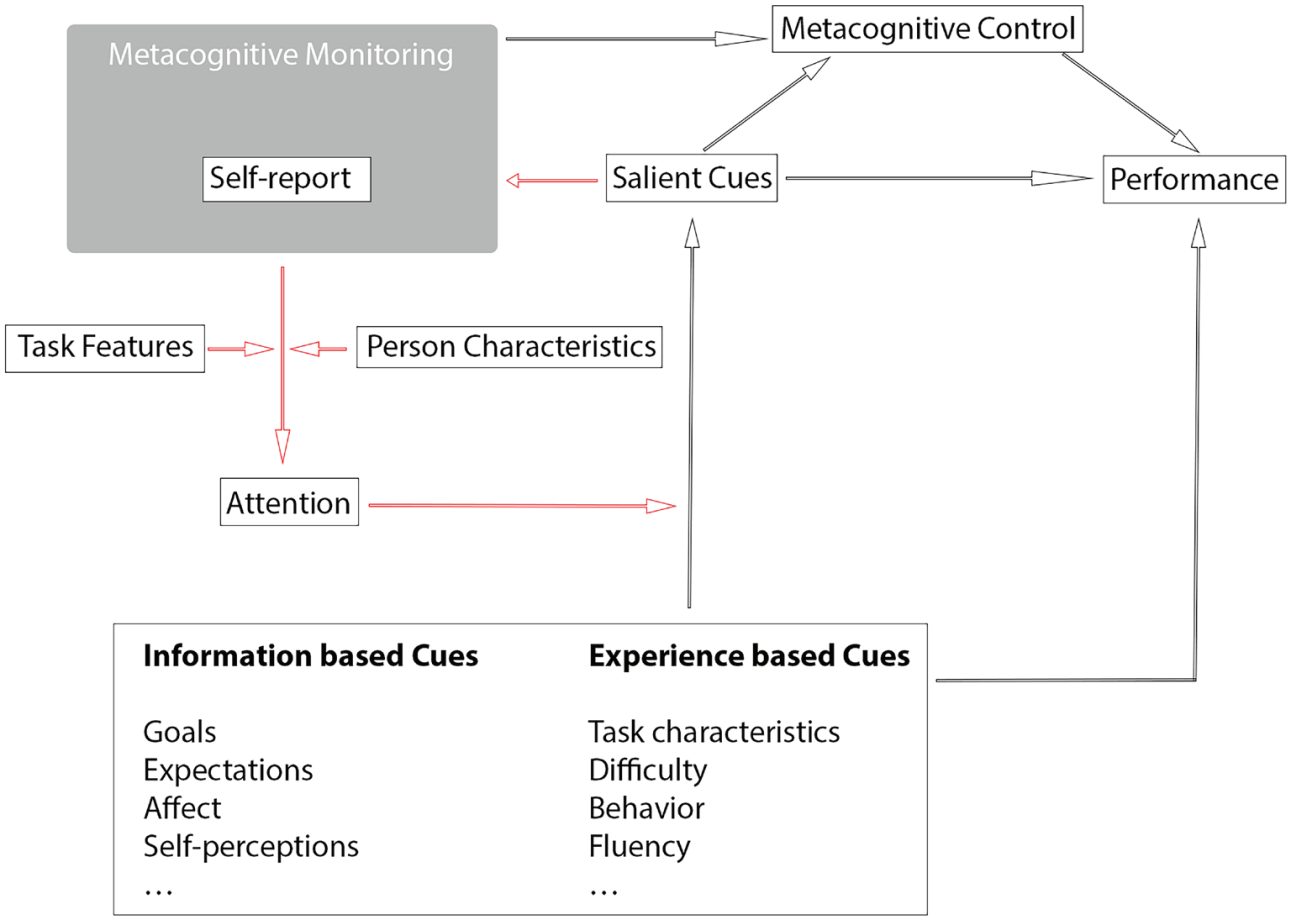
A Cue-driven Metacognitive Framework of Reactivity. Red paths indicate the additional demands that form the process of reactivity.

This model places the metacognitive self-report measure within the space of metacognitive monitoring, capturing some, but not all, of the monitoring process. This judgment is informed by salient cues, which are determined by a participant’s attention (Koriat et al., 2008). However, the inclusion of the self-report measure results in changes in the attentional system which lead to changes in the quantity and quality of cues that become salient (e.g., Soderstrom et al., 2015; Janes et al., 2018). In this way, the inclusion of a metacognitive self-report measure modifies the relationship between monitoring and control within the framework and that leads to changes in the salience of cues and ultimately changes in metacognitive monitoring and cognitive performance (red paths within Figure 1).

The extent to which the self-report measure modifies the attentional system is determined by task features (e.g., Mitchum et al., 2016) and person characteristics (Double and Birney, 2017a), as well as features of the self-report itself (Ericsson and Simon, 1993). This allows for the fact that some self-report measures of metacognition are relatively non-reactive because of their specific wording or framing (Fox et al., 2011; Double and Birney, 2019). It also allows for the fact that some tasks may be particularly reactive (Fox and Charness, 2010; Janes et al., 2018) and some people may be particularly prone to reactivity, for example participants with low self-confidence may be particularly affected by the evaluative nature of self-report judgments (Double and Birney, 2018).

Whether reactivity is positive or negative is predicted within this framework by the cues that become salient as a result of the inclusion of the self-report measure. While it is not possible to state absolutely whether attention to a particular cue will lead to positive or negative reactivity (and regardless, there are far too many potential cues to attempt to theorize for each one), two important determinants have been established by the literature thus far.

Firstly, the extent to which the cue is diagnostic of performance on the criterion test determines the direction of reactivity (Soderstrom et al., 2015; Janes et al., 2018). In general, when cues that are diagnostic of performance on the criterion test (i.e., cues that assist encoding/recall) are made salient due to the inclusion of the self-report, performance will improve. However, we hypothesize that when non-diagnostic (i.e., performance irrelevant) cues are made salient through the inclusion of a self-report, performance will be hindered, especially if these cues are processed at the expense of more diagnostic cues.

Secondly, the evidence suggests that some cues have a motivational effect on the goals and approach participants take toward the task by influencing their metacognitive control decisions. For example, Mitchum et al. (2016) found participants spend longer on easier items when JOLs are elicited and Double and Birney (2018) found that participants prioritize short-term over long-term performance when CR are elicited. Self-confidence may also fall into this category, because making confidence salient may motivate high self-confidence participants to persevere with harder items but deter low self-confidence participants. These changes in motivational and metacognitive control processes may be either conscious deliberate changes in behavior or unconscious changes resulting from the inclusion of the self-report measure.

This theoretical framework provides an explicit model of reactivity that focuses on attentional changes in cue processing, which have been previously proposed to account for reactivity (Soderstrom et al., 2015; Janes et al., 2018). This framework situates the study of reactivity within the broader model of metacognitive monitoring and control (e.g., Nelson and Narens, 1994), by integrating many of the recent findings which have examined *when* and for *whom* reactivity occurs. It is important to note that this model is tentative and is intended to help drive testable predictions, rather than stand as a finished product. This framework needs further expansion as there are likely to be additional pathways that need to be considered, for example the extent to which metacognitive self-reports place additional task demands on participants’ working memory, such as that proposed by a cognitive load approach.

## Implications of Reactivity

### Measurement Issue

The above review makes clear that reactivity may be a concern for researchers interested in metacognition. Notably, the results suggest that eliciting measures of metacognition might not only modify an individual’s underlying cognitive performance, but could also affect their metacognitive performance. The measures reviewed here (think-aloud protocols, JOLs, and CR) are the most widely used measures of metacognitive processes (Fleming and Lau, 2014), and a large amount of metacognitive research has relied on the assumption that these measures are an accurate method for measuring metacognition. The evidence for reactivity is a significant challenge for this assumption.

The potential reactivity of measures of metacognition may be particularly problematic because the magnitude and direction of reactivity appears to be inconsistent across participants, with some participants benefiting from reporting on their metacognition, whereas others are impaired or do not react (Fox and Charness, 2010; Double and Birney, 2019). Given these reactivity effects appear to vary systematically based on the cues used to make the judgment such as self-confidence and item difficulty, this may lead to systematic inaccuracies when assessing metacognition. A strong and consistent relationship between measures of metacognition and performance has been observed across the literature (Nelson and Dunlosky, 1991; Narens et al., 2008; Stankov et al., 2012; Stankov and Lee, 2014b). This correlation may, however, be inflated by the asymmetrical effect of providing a metacognitive self-report on participants’ performance. For example, if reporting one’s metacognition selectively enhances the performance of high self-confidence participants or performance on easy/related word-pairs, then trait confidence/difficulty is likely to be driving both the metacognitive rating and actual performance, as well as exaggerating the correlation between the two. Although further research is needed to confirm this hypothesis, it is noteworthy that correlations between metacognitive ratings and performance far exceed the correlation between “offline” self-report measures of confidence and performance (Stankov et al., 2012, 2014).

CR are perhaps the most common measure of consciousness, partially because they are grounded in a subjective sense of performance, rather than more “objective” measures such as post-decision wagering (Wierzchoń et al., 2012). While CR are often assumed to measure a subjective experience or a consciousness experience of performance (Wierzchoń et al., 2012), the fact that reactivity to CR is moderated by self-confidence (Double and Birney, 2017a, 2018, 2019) suggests that CR largely draw from pre-existing beliefs about confidence (i.e., information-based cues). In effect, the reactivity findings to date suggest that CR better reflect higher order mental representations of one’s competence, rather than a participant’s conscious experience of responding to the primary task. While both higher order beliefs and lower order subjective experience are aspects of consciousness, the use of CR as a measure of consciousness is problematic. This is because eliciting CR appears to direct attention toward some aspects of consciousness (and away from others), suggesting that CR cannot provide a “pure” measure of consciousness.

### Between-Group Artifacts

The findings reviewed here suggest that eliciting measures of metacognition may draw attention to salient cues. However, if the cue is part of an experimental manipulation, then eliciting metacognitive measures might artificially enhance experimental effects. This issue has been repeatedly shown with respect to pair relatedness, with performance difference between related and unrelated word-pairs heightened when JOLs are elicited (e.g., Mitchum et al., 2016). Additionally, Halamish (2018) recently found evidence that showing words in a very small font enhanced recall performance, but only when JOLs were not elicited. These findings are of critical importance because they suggest that researchers may be unintentionally directing attention to, and exaggerating the effect of, experimental manipulations because they are salient cues.

In addition, experimental manipulations may also interact with the inclusion of metacognitive self-reports for other reasons. Sidi et al. (2016) manipulated font disfluency between subjects either with or without eliciting CR. They observed that when CR were elicited, disfluency improved performance for screen-based problem-solving but hindered paper-based problem-solving; however, there was no effect when CR were not elicited. This finding suggests that even when experimental manipulations are not salient to participants (e.g., between-subject designs), the inclusion of metacognitive ratings can impact the results. More research is needed to examine the mechanisms in such cases and how they fit within the proposed model of reactivity.

### Metacognitive Decision-Making

More than simply being a nuisance to metacognitive researchers, studies of reactivity have also provided insights into the metacognitive process. The fact that metacognitive ratings are reactive under certain conditions suggests a number of implications about the nature and role of metacognitive monitoring for decision-making in cognitive tasks. In order for measures of metacognition to be reactive, they must presumably direct attention to information that participants would not otherwise attend to (Ericsson and Simon, 1993). Recent theories of cognitive control have posited that metacognitive judgments are used as the basis for decision-making in cognitive tasks (Ackerman, 2014). According to these models of metacognitive regulation, individuals decide to progress to the next item of a complex task when their confidence reaches a specific stopping criterion. These models argue that, at least for complex tasks, an individual’s stopping criterion is not fixed but rather diminishes gradually over time. Accordingly, as subjects spend more time on an item, they will gradually become more willing to accept a lower level of confidence (in the accuracy of their response) as a criterion for progressing to the next item. In support of such theories is the often-observed negative correlation between confidence and response time during cognitive tasks (Kelley and Lindsay, 1993; Ackerman and Koriat, 2011). However, it is possible that such stopping thresholds are in part a reaction to the demands of performing a metacognitive rating. For example, if measures of metacognition direct attention to self-confidence related beliefs, participants may be more likely to use their confidence as the basis for decision-making (metacognitive control) when metacognitive ratings are elicited compared to situations where they occur spontaneously.

The presence of reactivity also suggests that either the metacognitive processing elicited by self-report does not (necessarily) occur spontaneously when the metacognitive measure is not elicited or that the metacognitive process is, at least in part, an implicit process. Within consciousness research, CR are often used as a criterion for determining when information reaches a participant’s conscious awareness. For example, CR are often included in artificial grammar tasks to determine whether learning is above or below a threshold for conscious awareness (Norman and Price, 2015). However, eliciting CR may impact this subjective awareness itself by directing attention to various cues (Janes et al., 2018). For example, participants may become more aware of their learning by virtue of a self-report directing attention to learning-relevant cues (Berardi-Coletta et al., 1995), thereby effectively increasing their subjective awareness. The natural processes of subjective awareness of performance may therefore be substantially different to subjective awareness under conditions where CR are elicited (Double and Birney, 2018). This suggests that it may be problematic to use CR as a criterion for assessing subjective awareness, because this awareness may well be influenced by the elicitation of CR. One avenue that needs further exploration is the extent to which reactivity is compounded by eliciting simultaneous decision/CR. Tunney and Shanks (2003) argue that because subjective confidence is fleeting, researchers should elicit measures of confidence simultaneously with the decision/response, rather than as retrospective judgments. Given that even retrospective self-report ratings appear to influence participants’ performance, the simultaneous collection of responses and judgments may be even more problematic.

### Effect on Metacognition

The framework proposed here also suggests that eliciting measures of metacognition will influence metacognitive monitoring. According to the proposed framework, monitoring may be improved if the metacognitive measure directs attention, and makes salient, *performance-relevant* cues; however, if the metacognitive measure directs attention and makes salient *non-diagnostic* cues, monitoring accuracy may be impaired (Double and Birney, 2018). In terms of the measurement of metacognition, this has significant implications as it suggests that measures of metacognition disrupt the very processes that they are designed to assess. This also provides a strong impetus for interest in the specific wording and details of metacognitive ratings, because such details will influence the cues that participants utilize which may in turn affect their metacognitive monitoring, control, and ultimately their performance. For example, participants may attend to their trait like sense of self-confidence simply as the result of the word “confident” being present in a metacognitive measure (Double and Birney, 2019). Similarly, JOLs typically remind participants that they will be tested shortly, which may direct their attention to their beliefs about tests (such as test anxiety). In general, these findings suggest that researchers need to give greater consideration to the way they measure metacognition, because clearly the specific details of the measure matter in substantive ways.

### Cue Utilization

Reactivity research provides data about the cues that are utilized during the course of responding to various metacognitive measures. For example, the fact that pre-existing self-confidence moderates reactivity to CR suggest that CR direct attention to pre-existing self-confidence (and perhaps away from actual performance; see Double and Birney, 2018). This suggests that CR are utilizing information-based cues (pre-existing beliefs about one’s confidence) rather than experience-based cues (the subjective experience of solving an item). While this distinction has received substantial empirical study in the meta-memory literature (e.g., Koriat, 1997; Hertzog et al., 2013), this has not been the case with regard to CR, where it has typically been assumed that when individuals provide a confidence rating, they are utilizing experience-based cues to assess their performance on the previous item and not their self-perceptions (Ackerman, 2019). As such, the accuracy of CR may represent an individual’s trait self-confidence, rather than a micro-level assessment of their performance.

## Summary

Reactivity has been shown across a range of metacognitive self-report measures. The direction and magnitude of reactivity appear to be determined by a complex array of cues that are processed due to eliciting the metacognitive assessment. Here, we provided a review of the cues that may be driving reactivity and a theoretical account that integrates cue processing and cue salience to determine the impact of metacognitive self-reports on performance. Studying reactivity allows researchers to better understand how participants respond to metacognitive ratings, particularly which cues they draw on and how this may ultimately change their underlying cognitive processes. This suggests that metacognitive monitoring is driven by an attentional mechanism which directs attention toward salient cues. However, the qualities of the metacognitive measure (e.g., wording) may be muddying the waters by redirect metacognitive processes toward cues that would not ordinarily be processed as part of metacognitive monitoring, when self-report measures are not elicited.

## Future Directions

### What to Do About Reactivity?

Clearly measures of metacognition have provided valuable insights into how individuals monitor and control their own cognitive processes and researchers are very often interested in the effects of a manipulation (e.g., font size) on both cognitive performance and metacognition. When designing experiments that include measures of metacognition, researchers should be mindful of the potential effect of reactivity, which should involve (1) thinking carefully about the way in which metacognitive measures are elicited, and ideally (2) evaluating the effect of interest with and without the inclusion of metacognitive measures. With regard to designing measures that minimize reactivity, the current framework can provide some guidance. Firstly, the wording of the measure may need to be modified to avoid language that directs attention to information-based cues and even the scale may need to be considered (e.g., anchors). For example, some metacognitive measures use scales/anchors that are framed specifically as a probability estimate (e.g., “40% chance I responded correctly”), while other scales are centered around internal subjective feelings (e.g., “not confident at all”). The use of these different scales may direct attention to different cues and have different effects on performance. More research is needed to clarify what characteristics of metacognitive measures promote/negate reactivity, but for now researchers are advised to pay careful attention when designing measures of metacognition to try to limit the extent to which attention is unintentionally modified.

Of course, while there may be some modifications to the metacognitive measures themselves which may reduce reactivity to a level of no practical significance, there will presumably always remain some risk of any measure collected “online” at the time of responding being reactive. While researchers must of course weigh the risk of reactivity with pragmatic concerns, the best practice is to run experiments with and without the presence of measures of metacognition to ensure that effects generalize to both conditions. This seems particularly pertinent given that recent studies have shown that the significance of some experimental effects is contingent on the presence of metacognitive measures (Sidi et al., 2016; Halamish, 2018). However, it is worth noting that examining mean differences between a judgment condition and a control condition cannot rule out reactivity. Because reactivity is often moderated by other variables such as self-confidence, it is possible that the reactivity effect is obscured by these moderations. For example, Double and Birney (2017a) found that there was no difference between mean performance in a confidence rating group compared to a control, but this was due to the fact that performance of high self-confidence individuals improved, while performance of low self-confidence individuals was impaired. As yet, there does not appear to be any easy solution to ruling out reactivity effects completely.

### Monitoring Accuracy

Another question that remains unanswered by the current research is the extent to which metacognitive accuracy modifies reactivity. There exist significant individual differences in both metacognitive monitoring accuracy (Jackson and Kleitman, 2014) and the extent to which individuals naturally monitor their own performance (Pirolli and Recker, 1994). It is plausible then that participants who do not tend to monitor their performance reliably or accurately will be more affected by performing measures of metacognition, because they would not have otherwise attended to their subjective beliefs about their performance. As most studies of reactivity have a control group that does not provide ratings, it is difficult to examine the moderating effects of metacognitive ability directly. A study by Double and Birney (2019) was however able to tentatively address the question because the control group performed likelihood ratings (i.e., CR with the work “confident” replaced by the word “likely”). While our study did not find a significant moderation of reactivity as a function of metacognitive monitoring ability (measure through within-subject gamma correlations), the effect approached significance and the study was somewhat underpowered to address this question (as it was not the primary aim of the study).

### Long-Term Effects

A somewhat open question within the reactivity literature is to what extent the changes in recall are longer lasting? As mentioned previously, the demand to self-assess prompted by measures of metacognition may focus participants on short-term performance, which may or may not come at the expense of long-term learning (Double and Birney, 2017b). Witherby and Tauber (2017) performed one of the only studies of the long-term effects of eliciting JOLs on recall. In a series of experiments, they found that eliciting JOLs during the presentation of related word-pairs improved both short-term (3-min) and long-term (2 days) retention. The long-term effects are particularly important from an applied perspective as metacognitive prompting is commonly used in classrooms and online learning environments to facilitate improved learning (Yarrow and Topping, 2001; Bannert, 2006; Bannert et al., 2009; Peters and Kitsantas, 2010; Double and Birney, 2016; Colmar and Double, 2017). The use of more domain-general metacognitive self-report measures (such as think-aloud protocols) in learning and educational environments appears to be a promising avenue, although this needs to be considered against the fact that some learners may be negatively impacted (e.g., low self-confidence learners).

### Emotion, Anxiety, and Individual Differences

In looking at person-level moderators of reactivity, research has tended to draw on a social-cognitive framework (Bandura, 1982, 1986, 2001) of motivational goals and self-regulation, when considering the impact the act of providing a metacognitive rating has on cognitive performance. However, reactivity triggered by experience-based cues implicates a potential role for a broader range of individual differences moderators. Likely candidates for further investigations include anxiety (emotional stability), because of the known effects on cognitive performance (e.g., test anxiety) and emotional processing generally, as well as age, gender, and cultural backgrounds. If reactivity cannot be controlled at a task level, then it is important to understand who is most susceptible to such effects and under what conditions, particularly if those effects are negative.

## Conclusions

Self-report measures are often utilized to assess the effectiveness of an individual’s metacognitive abilities. However, the implicit assumption underlying this approach is the idea that eliciting these measures from individuals does not alter the processes that they were designed to measure. The recent research concerning reactivity has challenged this assumption by demonstrating that participants do in fact react to performing measures of metacognition. Perhaps more problematically from a measurement perspective, it appears that individuals react to providing these measures in systematically different ways. Additionally, there is evidence that such measures may direct attention to salient cues and may be exaggerating the effect of experimental manipulations. This research therefore provides a substantial challenge to the dominant measurement paradigm in metacognition research. By providing a framework for the study of reactivity, we hope to encourage better understanding of how individuals react when measures of metacognition are elicited and point to the importance of constructing measures that minimize reactivity, as well as measuring reactivity effects.

More optimistically, reactivity research has provided insights into the cues utilized when participants make different metacognitive ratings and the fact that such metacognitive self-assessment may not naturally occur when metacognitive measures are not elicited, suggesting that we may not consciously monitor our performance when ratings are not elicited in the same way as when they are. Further work is needed not simply to inform researchers about how they can minimize reactivity, but to further explore what reactivity can tell us about metacognitive processes and consciousness.

## Author Contributions

KD prepared a draft of this manuscript. DB provided feedback and reviewed the manuscript.

### Conflict of Interest

The authors declare that the research was conducted in the absence of any commercial or financial relationships that could be construed as a potential conflict of interest.
